# Pulsed-field ablation versus radiofrequency ablation in patients undergoing repeat catheter ablation for atrial fibrillation

**DOI:** 10.1016/j.hroo.2025.07.002

**Published:** 2025-07-08

**Authors:** Corinne Isenegger, Gianluca Di Bari, Rebecca Arnet, Fabian Jordan, David Spreen, Behnam Subin, Philipp Krisai, Sven Knecht, Nicolas Schaerli, Gian Voellmin, Felix Mahfoud, Christian Sticherling, Michael Kühne, Patrick Badertscher

**Affiliations:** 1 Department of Cardiology, University Hospital Basel, Basel, Switzerland; 2 Cardiovascular Research Institute Basel, University Hospital Basel, Basel, Switzerland

**Keywords:** Atrial fibrillation, Pulsed-field ablation, Radiofrequency ablation, Pulmonary vein isolation, Catheter ablation

## Abstract

**Background:**

Pulsed-field ablation (PFA) is noninferior compared with thermal energy sources such as radiofrequency ablation (RFA) or cryoballoon ablation for de novo pulmonary vein isolation (PVI). However, its potential value for repeat catheter ablation (CA) after unsuccessful thermal PVI has not been assessed.

**Objective:**

This study aimed to compare the procedural characteristics, safety, and efficacy between PFA and RFA in patients undergoing repeat CA after previous thermal PVI.

**Methods:**

Patients with recurrent atrial fibrillation (AF) undergoing repeat CA at a tertiary referral center were prospectively enrolled. All redo procedures were guided by a 3-dimensional electroanatomic mapping system and performed using either PFA or RFA.

**Results:**

A total of 185 patients underwent repeat CA for AF (median age 68 years [60–74], 31% female, 53% paroxysmal AF, and 47% persistent AF). PFA was used in 71 patients (38%), whereas RFA was used in 114 patients (62%). The median procedure time was similar between the PFA and RFA groups (61 minutes [50–71] vs 56 minutes [45–82] *P* = .956), whereas fluoroscopic time was longer in the PFA group (8 minutes [6–12] vs 4 minutes [2–8], *P* < .001). A total of 4 complications occurred, all in the RFA group. Recurrence-free survival rates after repeat CA were 61% and 69% in the PFA vs the RFA group after a follow-up of 365 days (*P*_log-rank_ = .082). Posterior wall isolation was more commonly performed in the PFA group (72% vs12%).

**Conclusion:**

In patients undergoing repeat CA after thermal PVI, PFA resulted in similar procedural characteristics, safety, and arrythmia-free survival compared with RFA.

## Graphical abstract

**Figure:**
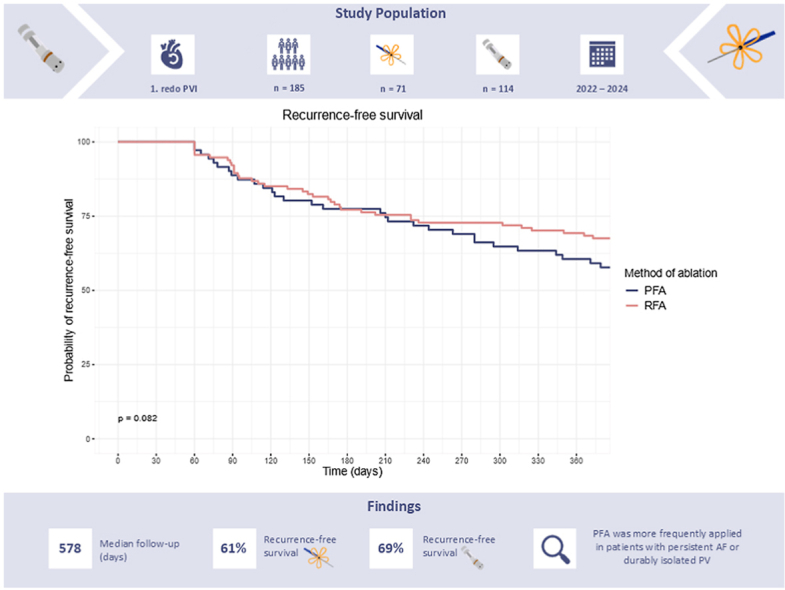

**Key Findings**

▪ Arrhythmia-free survival was comparable between the pulsed-field ablation (PFA) and radiofrequency ablation (RFA) groups undergoing repeat catheter ablation for atrial fibrillation.
▪ Procedure time and left atrial dwell time were similar, but fluoroscopy time was longer in the PFA group.
▪ Posterior wall isolation was performed in 4 of 5 patients in the PFA group compared with only 1 of 5 in the RFA group.

## Introduction

Pulmonary vein isolation (PVI) is an effective treatment option for patients with atrial fibrillation (AF). Recent randomized controlled trials have demonstrated the superiority of catheter ablation (CA) by PVI over antiarrhythmic therapy for the treatment of AF.^1^, ^2^, ^3^ However, despite advancements in energy sources and ablation techniques, AF recurs in approximately 30% of patients.^4^, ^5^, ^6^, ^7^

Although current guidelines provide clear recommendations for de novo ablation procedures, there is a notable lack of studies and recommendations for redo CA procedures. Although PV reconnection is widely accepted as the primary cause of AF recurrence, the role of structural disease progression or the emergence of non–pulmonary vein (PV) triggers is less well studied and requires further investigation.

Recently, pulsed-field ablation (PFA) has been introduced and quickly became a commonly used energy source for CA of AF^6^^,^^8^, ^9^, ^10^ owing to its favorable safety profile and high rates of durable PVI.^7^ However, data on the use of PFA in repeat CA of AF, including ablation strategies beyond PVI such as posterior wall isolation (PWI), are currently lacking.

A currently available pentaspline PFA catheter is optimized for PVI, but is also a very well-suited tool for PWI. A recent study reported the early experience of PWI using PFA and demonstrated a high rate of successful PWI in an efficient and safe manner using the pentaspline PFA catheter.^11^ PWI remains an important area of study for repeat CA, particularly because it may be a key source of non-PV triggers.

This study aimed to compare procedural characteristics, safety, and efficacy of PFA and RFA in patients undergoing repeat CA after previous thermal PVI.

## Methods

### Patient population

The study population consisted of adult patients with paroxysmal or persistent AF undergoing their first repeat CA at the University Hospital Basel, Switzerland, between January 2022 and February 2024. These patients were consecutively enrolled in the prospective SWISS-AF-PVI registry (NCT03718364). For this study, only patients with thermal energy sources used for the index procedure were included. Ablation procedures using PFA or unknown energy sources during the index procedure were excluded. The patients were divided into 2 groups based on the ablation methods used at the first repeat CA: PFA and RFA. The ablation method was selected at the discretion of the treating electrophysiologist. All patients had to be at least 18 years of age and provide a written informed consent. The study was performed according to the principles of the Declaration of Helsinki and was approved by the local ethics committees.

### Procedures

#### Preprocedural management

Before the procedure, all patients underwent transesophageal echocardiography to exclude the presence of a left atrial (LA) thrombus. Furthermore, each patient underwent preprocedural imaging using either computed tomography or magnetic resonance imaging of the LA if not already obtained during the index procedure.

#### PVI

Five experienced electrophysiologists conducted the interventions. The sedation was performed under conscious sedation with midazolam, fentanyl, and propofol. In all cases, femoral access was obtained with ultrasound guidance. Transseptal puncture was performed using fluoroscopic guidance or, in selected cases, intracardiac echocardiography. To maintain the activated clotting time at a targeted value of 350 seconds, intravenous heparin was used. Intracardiac electrograms and surface electrograms were recorded at a speed of 100 mm/s.

#### Mapping

Pre- and postablation voltage mapping was performed using a 3-dimensional electroanatomic mapping (3D-EAM) system (CARTO 3, Biosense Webster, Irvine, CA) in combination with a multipolar mapping catheter (Pentaray or Octaray, Biosense Webster). Preablation mapping was used to assess PV anatomy and identify PV reconnections or extra-PV low-voltage areas when present, although systematic provocation of non-PV triggers was not performed. In cases of incomplete or ostial PVI, the PFA group received the standard 8 applications per PV, whereas segmental reisolation in the RFA group was guided by a high-power short-duration (HPSD) protocol.^12^ Extra-PV lesion sets, including linear lesions such as the mitral isthmus or cavotricuspid isthmus (CTI), were added at the treating physician’s discretion. In such cases, postablation mapping was used to confirm the acute success of each lesion set, including verification of bidirectional conduction block across linear ablation lines using standard pacing techniques.

#### PFA

The FARAPULSE PFA system (FARAPULSE, Boston Scientific, Natick, MA) is a medical device comprising a custom generator, which delivers a pulsed electrical waveform over multiple channels (Farastar), a 13-F steerable sheath (Faradrive), and a 12-F over-the-wire PFA catheter (FaraWave). Each of the catheter’s 5 splines is equipped with 4 electrodes. After the transseptal puncture, a J-tip guidewire was used to cannulate the PVs, after which the 31-mm FaraWave catheter (Boston Scientific, Marlborough, MA) was introduced into the LA. The device can be configured into 2 distinct poses: the flower and basket configuration. Redo PVI was performed with 4 applications in each configuration per vein. A rotation of 30° to 40° was applied between each pair of PFA applications, after the 2 initial applications in each configuration. The ablation procedure was conducted at a voltage of 2 kV.

PWI was conducted as previously described.^11^ In brief, using the catheter in flower configuration, 4 anchor lesions per vein extending to the LA PW were deployed. Then, the wire was pulled back into the catheter, and the PFA catheter was rotated along the entire LA PW in a stepwise fashion using the flower configuration to create overlapping lesions. This was guided by the 3D-EAM system and by visualizing the ablation catheter. After PWI, a detailed 3D-EAM was again generated, and additional PFA lesions were applied to complete PVI and PWI as necessary. Other ablation strategies included application of an anterior mitral isthmus line in flower configuration by connecting the mitral annulus to the right superior PV and substrate modification of the anterior wall in flower configuration.

#### Radiofrequency ablation

RFA was performed using the previously described HPSD protocol.^12^ Ablation was performed at the anatomic ostium defined by the 3D-EAM using the ablation index with a minimal target value of 400 at the posterior wall and 450 at the anterior wall.^12^ Interlesion distance was set to 6 mm. For the HPSD protocol (HPSD group), power was set to 45–50 W at the posterior wall and the anterior wall. The ThermoCool SmartTouch SF (Biosense Webster) ablation catheter with a flow rate of 8–15 mL/min was used to perform all ablations. Lesions were applied only when stable catheter-tissue contact was confirmed, and ablation index thresholds guided lesion quality. Additional lesions, such as application of a box lesion or roof line, mitral isthmus line including vein of Marshall ethanol ablation, CTI line, and isolation of the superior vena cava, were at the discretion of the treating physician and were performed as previously described.^13^

#### Postablation management

Oral anticoagulation therapy was continued at a minimum of 2 months after ablation. A figure-of-8 suture was used to establish homeostasis, which was subsequently followed by a 4- to 6-hour bed rest period. Transthoracic echocardiography within 1 hour of the procedure was performed to exclude pericardial effusion.

### Biomarkers of myocardial injury

Blood samples were obtained in a fasting state on the morning before the procedure and 24 hours after the procedure. An assay for high-sensitivity cardiac troponin T (hs-cTnT) was used (Roche Elecsys 2010 high-sensitivity troponin T, Roche Diagnostics) with a 99th percentile concentration of 14 ng/L and a corresponding coefficient of variation of 10% at 13 ng/L.^14^

### End points and follow-up

The primary efficacy outcome of the study was arrhythmia-free survival during follow-up. After a blanking period of 60 days,^15^ any atrial arrhythmia, such as AF, typical atrial flutter, or atypical atrial flutter with a duration of at least 30 seconds, was defined as recurrence. In the event of recurrence occurring within the blanking period and persisting beyond 60 days, the date of the recurrence was set to the end of the blanking period. Secondary efficacy endpoints included the total duration of the procedure (ie, the period from femoral puncture to sheath removal) and the duration of LA dwell time. The safety endpoints included phrenic nerve injury, pericardial tamponade, transient ischemic attack/stroke, and vascular access complications. The scheduled follow-up involved outpatient clinic visits after 3, 6, and 12 months. The visits consisted of a physical examination, 12-lead electrocardiogram (ECG), and 1- to 7-day Holter-ECG. The same follow-up was provided to patients with implantable cardiac devices. In the case of loop recorders, remote monitoring was used for arrhythmia detection. In the event of symptomatic recurrence outside the planned follow-up, a 12-lead ECG and/or a Holter ECG was recorded for arrhythmia documentation.

### Statistical analysis

Continuous variables are described using the median and interquartile range and compared using the Wilcoxon rank-sum test. Categorical variables are presented as numbers and percentages and compared using either the χ^2^ or Fisher’s exact test, as appropriate. The test of proportions was used to compare recurrences. The Kaplan-Meier analysis, together with the log-rank test, was used to compare the probability of freedom from atrial arrhythmia recurrence*. P* < .05 was considered statistically significant. The analysis was performed using R (R Core Team [2021], R Foundation for Statistical Computing, Vienna, Austria) and RStudio 2023.09.1 (RStudio Team [2019], RStudio, Inc, Boston, MA).

## Results

### Baseline characteristics

A total of 185 patients underwent repeat CA. The median age was 68 years (60–74), and 31% were female. The median left ventricular ejection fraction was 58% (53%–62%), and the median LA volume index was 38 mL/m^2^ (31–45). The primary reasons for repeat CA were paroxysmal AF in 51%, persistent AF in 43%, and atypical atrial flutter in 9 patients (5%). PFA was used in 71 patients (38%) and RFA in 114 patients (62%). Baseline characteristics were similar between the PFA and RFA groups (Table 1). The energy source used for the index PVI did not differ between the groups: RFA was used in 42 patients (59%) vs 71 patients (62%), and CA energy in 29 patients (41%) vs 43 patients (38%) in the PFA and the RFA groups, respectively (*P* = .788).

**Table 1:** Patient characteristics of patients undergoing a PFA and RFA for PVI

Variable	Overall, N = 185	PFA, n = 71	RFA, n = 114	*P* value
Age, y	68 [60–74]	67 [61–75]	69 [60–74]	.849
Sex (female)	57 (31%)	22 (31%)	35 (31%)	.968
BMI, kg/m^2^	26 [24–30]	27 [25–31]	26 [23–29]	.011
AF type				.003
Paroxysmal	97 (53%)	28 (39%)	69 (62%)	
Persistent	86 (47%)	43 (61%)	43 (38%)	
CHADS_2_-VASc score	2 [1–3]	2 [1–3]	2 [1–3]	.821
LA diameter, mm	40 [37–45]	41 [37–46]	40 [36–44]	.089
LAVI, mL/m^2^	38 [31–45]	40 [34–44]	37 [28–46]	.217
LVEF %	58 [53–62]	57 [50–61]	59 [55–64]	.026
Coronary artery disease	16 (9%)	7 (10%)	9 (8%)	.644
Hypertension	113 (61%)	48 (68%)	65 (57%)	.151
Hypercholesterinemia	54 (37%)	26 (45%)	28 (32%)	.111
Diabetes	12 (6%)	7 (10%)	5 (4%)	.218
Smoking history	85 (49%)	36 (54%)	49 (46%)	.308
AAD	43 (23%)	14 (20%)	29 (25%)	.370
Type of AAD				.399
Amiodarone	23 (53%)	8 (57%)	15 (52%)	
Dronedarone	3 (7%)	1 (7%)	2 (7%)	
Flecainide	13 (30%)	3 (21%)	10 (34%)	
Propafenone	1 (2%)	1 (7%)	0 (0%)	
Sotalol	2 (5%)	0 (0%)	2 (7%)	
Verapamil	1 (2%)	1 (7%)	0 (0%)	
Available imaging				.850
CT	22 (12%)	8 (12%)	14 (13%)	
MR	156 (88%)	60 (88%)	96 (87%)	

*P* values were calculated using the Wilcoxon rank-sum, Pearson’s χ^2^, or Fisher’s exact test, as appropriate.

AAD = antiarrhythmic drug; AF = atrial fibrillation; BMI = body mass index; CT = computed tomography; LA = left atrial; LAVI = left atrial volume index; LVEF = left ventricular ejection fraction; MR = magnetic resonance; PFA = pulsed-field ablation; RFA = radiofrequency ablation.

### Procedural characteristics

Median procedure duration and LA dwell time were similar between the PFA and RFA groups: 61 minutes (50–71) vs 56 minutes (45–82) (*P* = .956) and 41 minutes (32–52) vs 41 minutes (31–63) (*P* = .623), respectively. Fluoroscopy time was significantly longer in the PFA group than the RFA group with 8 minutes (6–12) vs 4 minutes (2–8) (*P* < .001). Among patients who only underwent redo PVI without additional lesions, the median procedure time, LA dwell time, and fluoroscopy time were 49 minutes (43–60) vs 49 minutes (40–56) (*P* = .910), 31 minutes (26–36) vs 33 minutes (26–42) (*P* = .451), and 8 minutes (6–10) vs 4 minutes (2–7) (*P* < .001), respectively, in the PFA and RFA groups.

Most patients (74%) had at least 1 reconnected PV, but the number of reconnected PV differed between the 2 groups: all PVs were isolated in 31 patients (44%) vs 16 patients (14%) in the PFA vs RFA groups (*P* < .001), whereas only 1 reconnected PV was found in 20 patients (29%) vs 43 patients (38%) (*P* = .267) and ≥2 reconnected PVs in 19 patients (27%) vs 55 patients (48%) (*P* = .007), respectively. Acute redo PVI was achieved in 100% of patients in both groups. hs-cTnT measured 1 day after the procedure was significantly higher in the PFA group than the RFA group: 833 ng/L (562–1068) vs 325 ng/L (166–513) (*P* < .001). Procedural characteristics are presented in Table 2.

**Table 2:** Comparing procedural characteristics of PVI using PFA and RFA

Variable	Overall, N = 185	PFA, n = 71	RFA, n = 114	*P* value
Procedure duration, min	59 [47–77]	61 [50–71]	56 [45–82]	.956
LA dwell time, min	41 [31–58]	41 [32–52]	41 [31–63]	.623
Fluoroscopy time, min	7 [3–10]	8 [6–12]	4 [2–8]	<.001∗
Fluoroscopy dose, Gy cm^2^	383 [165–886]	469 [250–945]	333 [105–620]	.004∗
Ablation duration, min	17 [10–30]	14 [9–20]	19 [10–34]	.035∗
Mapping duration, min	13 [9–19]	14 [11–19]	12 [9–19]	.236
Rhythm before ablation				.003∗
AF	52 (28%)	29 (41%)	23 (20%)	
AT/AFlu	19 (10%)	3 (4%)	16 (14%)	
SR	113 (61%)	38 (54%)	75 (66%)	
Ablation method of index PVI				.788
Cryo	72 (39%)	29 (41%)	43 (38%)	
RFA	113 (61%)	42 (59%)	71 (62%)	
Number of reconnected veins				<.001∗
0	47 (26%)	31 (44%)	16 (14%)	
1	63 (34%)	20 (29%)	43 (38%)	
≥2	74 (40%)	19 (27%)	55 (48%)	
Reconnected LIPV	34 (18%)	8 (11%)	26 (23%)	.083
Reconnected LSPV	49 (27%)	16 (23%)	33 (29%)	.462
Reconnected RIPV	76 (41%)	14 (20%)	62 (54%)	<.001∗
Reconnected RSPV	80 (43%)	25 (35%)	55 (48%)	.112
Hs-cTnT before PVI, ng/L	10 [7–16]	11 [7–16]	10 [7–15]	.638
Hs-cTnT 1 d after PVI, ng/L	456 [225–825]	833 [562–1068]	325 [166–513]	<.001∗

The ablation duration is defined as the time from the first ablation to the last ablation. *P* values were calculated with the Wilcoxon rank-sum test, 2-sample test for equality of proportions, or Pearson’s χ^2^ test, as appropriate.

AF = atrial fibrillation; AFlu = atrial flutter; AT = atrial tachycardia; Cryo = cryoballoon ablation; Hs-cTnT = high-sensitive cardiac troponin T; LA = left atrial; LIPV = left inferior pulmonary vein; LSPV = left superior pulmonary vein; PFA = pulsed-field ablation; PVI = pulmonary vein isolation; RFA = radiofrequency ablation; RIPV = right inferior pulmonary vein; RSPV = right superior pulmonary vein; SR = sinus rhythm.

∗ Statistically significant.

### Additional lesion sets beyond redo PVI

Additional lesions beyond redo PVI were applied in 108 patients (58%), significantly more often in the PFA group than the RFA group (77% vs 46%, *P* < .001). In the PFA group, the most common lesion set was PWI in 51 patients (93%), followed by substrate modification in 8 patients (11%), lateral mitral isthmus lines in 3 patients (4%), and CTI ablation in 5 patients (7%). In the RFA group, PWI or roof line was performed in 23 patients (43%), 15 patients (13%) received a lateral mitral isthmus line, 8 patients (7%) underwent vein of Marshall ethanol ablation, 11 patients (10%) underwent substrate modification, 5 patients (4%) had superior vena cava isolation, and 23 patients (20%) underwent CTI ablation. Additional lesion sets are presented in Table 3.

**Table 3:** Additional lesions of patients undergoing catheter ablation for AF using PFA or RFA

Variable	PFA, n = 71	RFA, n = 114	*P* value
Posterior wall isolation or roof line	51 (93%)	23 (43%)	<.001∗
Mitral isthmus line	3 (4%)	15 (13%)	.046∗
Cavotricuspid isthmus ablation	5 (7%)	23 (20%)	.015∗
Vein of Marshall ethanol ablation	0 (0%)	8 (7%)	.024∗
Superior vena cava isolation	0 (0%)	5 (4%)	.158
Extra PVI foci	3 (4%)	1 (1%)	.158
Substrate modification	8 (11%)	11 (10%)	.724

*P* values were calculated using Pearson’s χ^2^ test or Fisher’s exact test, as appropriate.

AF = atrial fibrillation; PFA = pulsed-field ablation; PVI = pulmonary vein isolation; RFA = radiofrequency ablation.

∗ Statistically significant.

### Procedural safety

Overall, 4 complications (3%) occurred, all in the RFA group. These included 2 cases of cardiac tamponade treated with percutaneous drainage, 1 allergic reaction, and 1 major access site complication requiring surgical intervention (Table 4).

**Table 4:** Safety and efficacy in patients undergoing PFA or RFA

Variable	Overall, N = 185	PFA, n = 71	RFA, n = 114	*P* value
Recurrence	83 (45%)	37 (52%)	46 (40%)	.118
Type of recurrence				.282
AF	63 (76%)	26 (70%)	37 (80%)	
AT	20 (24%)	11 (30%)	9 (20%)	
Use of AAD	12 (6%)	7 (10%)	5 (4%)	.218
Type of AAD				.197
Amiodarone	11 (6%)	6 (8%)	5 (4%)	
Flecainide	1 (1%)	1 (1%)	0 (0%)	
None	173 (94%)	64 (90%)	109 (96%)	
Complications	4 (2%)	0 (0%)	4 (4%)	.300
Type of complication				.819
Allergic reaction	1 (1%)	0 (0%)	1 (1%)	
Bleeding required surgical treatment	1 (1%)	0 (0%)	1 (1%)	
Tamponade	2 (1%)	0 (0%)	2 (2%)	
Median follow-up, d	578 [212–840]	483 [210–700]	661 [230–932]	.012∗

*P* values were calculated using a 3-sample test for equality of proportions, Pearson’s χ^2^ test, Fisher’s exact test, or the Wilcoxon rank-sum test, as appropriate.

AAD = antiarrhythmic drug; AF = atrial fibrillation; AT = atrial tachycardia; PFA = pulsed-field ablation; RFA = radiofrequency ablation.

∗ Statistically significant.

### Arrhythmia-free survival and follow-up

**Figure 1:**
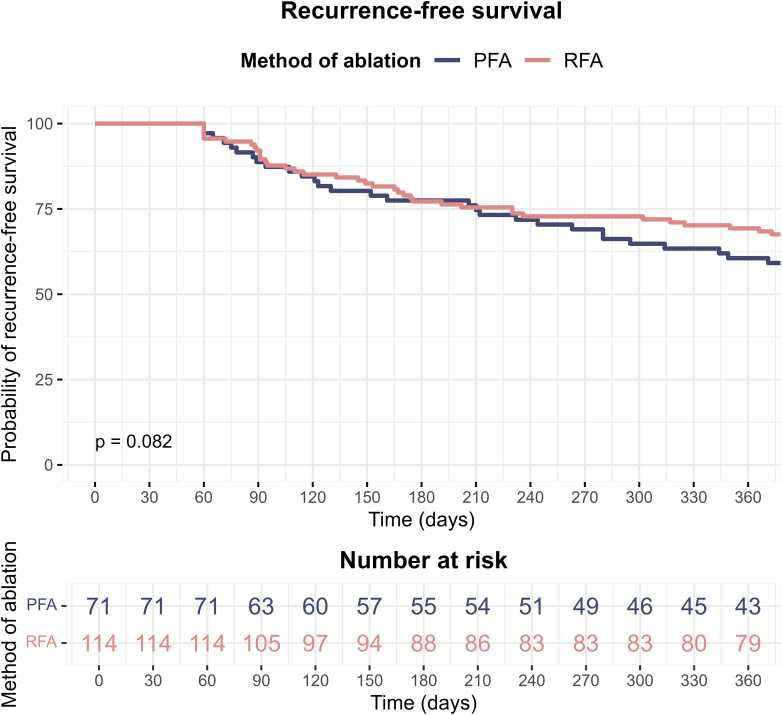
Kaplan-Meier curve comparing the probability of recurrence-free survival over 1 year between the PFA group and the RFA group. PFA = pulsed-field ablation; RFA = radiofrequency ablation.

During a median follow-up of 365 days, the estimated Kaplan-Meier curve (Figure 1) showed a recurrence-free survival rate of 61% (95% confidence interval [CI] 50–73) for the PFA group vs 69% (95% CI 61–78) for the RFA group (*P*_log-rank_ = .082).

Within the PFA and RFA groups, the recurrence of AF was observed in 26 patients (70%) vs 37 patients (80%), and atrial tachycardia or atrial flutter in 11 patients (30%) vs 9 patients (20%). Among patients with recurrence, 73% (PFA) and 78% (RFA) were symptomatic (Supplemental Figure 1, Supplemental Table 1). Recurrences were primarily detected using 12-lead ECG (39%), Holter monitoring (24–48 hours 35%, 4–7 days 10%), and implantable loop recorders (11%), with no significant differences between the PFA and RF groups (*P* = .715).

In a subgroup analysis of patients with all PVs isolated (n = 77, 16 in the PFA group [21%] and 61 in the RFA group [79%]), the recurrence-free survival rates were 42% (95% CI 28–63) for the PFA group vs 50% (95% CI 31–82) for the RFA group (*P*_log-rank_ = .358) (Supplemental Figure 2). When only comparing patients with ≥1 reconnected PV (n = 137, 39 in the PFA group [28%] and 98 in the RFA group [72%]), the recurrence-free survival rates were 77% (95% CI 65–91) for the PFA group vs 72% (95% CI 64–82) for the RFA group (*P*_log-rank_ = .691) (Supplemental Figure 3). Among patients with persistent AF (n = 86, 43 in the PFA group [61%] and 43 in the RFA group [38%]), the recurrence-free survival rates were 58% (95% CI 45–75) vs 63% (95% CI 50–79) (*P*_log-rank_ = .742) (Supplemental Figure 4). Further information about the subgroup analysis of patients with persistent AF is presented in Supplemental Table 2.

## Discussion

This large study compared the efficiency, safety, and efficacy of 2 different ablation modalities in patients undergoing repeat CA of AF after a previous thermal PVI. The key findings are the following:

First, the arrhythmia-free survival was comparable between the groups. This was also found for patients with persistent AF and those with durably isolated PVs. Second, procedural metrics such as procedure time and LA dwell time were similar between groups, whereas fluoroscopic time was longer in the PFA group. Third, low complication rates were observed, demonstrating high safety for both modalities. Fourth, PFA was the preferred modality for patients with persistent AF or with durably isolated PVs. PWI was performed in 4 of 5 patients in the PFA group, but only in 1 of 5 in the RFA group.

These findings highlight the advantages and limitations of PFA compared with RFA, particularly in the context of repeat CA of AF. Previous studies have shown that PFA is generally associated with shorter procedure times than RFA.^7^^,^^16^, ^17^, ^18^ However, in our study, procedure times were similar. One may speculate that this is related to the higher frequency of additional lesions beyond redo PVI in the PFA group, which is also reflected in the significantly higher hs-cTnT levels observed in the PFA group.

Both PFA and RFA achieved a 100% success rate for acute PVI, consistent with previous studies reporting success rates of 99%–100%.^7^^,^^12^^,^^16^^,^^19^ However, data comparing PFA and RFA for repeat CA of AF are limited. A recently published study by Dello Russo et al^17^ compared PFA (n = 192) and very-high-power short-duration (vHPSD) RFA (n = 368) as modalities for AF treatment in both first and redo CA. Among the patients undergoing a repeat procedure, 1-year Kaplan-Meier estimates showed a recurrence-free survival of 88% in the PFA vs 61% in the vHPSD group (*P*_log-rank_ = .033). However, when only patients without class I/III antiarrhythmic drugs were included, the difference between PFA and vHPSD diminished (63% vs 57%, *P*_log-rank_ = .67). Another study by Gunawardene et al^16^ focused on PFA vs RFA for atrial tachycardia ablation after previous AF ablation. Among the 32 patients receiving PFA and the 35 patients receiving RFA, the PFA group showed a lower estimated arrhythmia-free survival rate (63%) than the RFA group (87%) after a 1-year follow-up. Similarly, limited data exist on other single-shot devices for repeat CA. For example, Pokushalov et al^20^ conducted a small randomized trial comparing a single-shot device (CA) vs RFA for repeat CA in 80 patients, reporting a 1-year recurrence-free survival of 58% for RFA and 43% for cryoablation.

Herein, the recurrence-free survival rate was similar between the PFA and RFA groups, despite a higher proportion of patients with persistent AF and more durable isolated PVs in the PFA group. Whether the outcome of the PFA group would have been worse without additional lesions remains speculative, but our findings indicate that PWI is more likely to be performed when using PFA. Currently, there are no randomized data regarding the value of PWI on top of PVI when using PFA. The PIFPAF-PFA trial (NCT05986526) will randomize patients with persistent AF toward PVI only vs PVI and PWI outside the repeat CA population and provide important insights.

### Limitations

Several limitations should be acknowledged. First, this is a nonrandomized, observational, single-center study with inherent limitations. It is particularly important to highlight the small differences in baseline characteristics of the groups and the variation in group sizes. Second, the statistical power of the study is limited. Nevertheless, to the best of our knowledge, this is the largest study comparing PFA with RFA for repeat CA after thermal PVI. Third, not all patients had continuous monitoring with implantable loop recorders, which may have influenced the detection rates of arrhythmia recurrence.

## Conclusion

In patients undergoing repeat CA after thermal PVI, PFA results in similar procedural characteristics, safety, and arrythmia-free survival compared with repeat RFA. PFA was more frequently used in patients with persistent AF or durably isolated PV. Additional PWI was performed in 4 of 5 patients in the PFA group compared with 1 of 5 in the RFA group. Further randomized trials assessing the role of PFA for repeat CA are warranted.
