# Supplementary material for: Pulsed-field ablation versus radiofrequency ablation in patients undergoing repeat catheter ablation for atrial fibrillation
Source: Heart Rhythm O2. 2025 Jul 8;6(10):1483–90. doi: 10.1016/j.hroo.2025.07.002 (PMC12570212; doi:10.1016/j.hroo.2025.07.002)
Supplement: Supplementary Appendix [file mmc1.docx]

Supplementary Appendix

Table of contents

[Figure S1 2](#_Toc199609223)

[Figure S2 3](#_Toc199609224)

[Figure S3 4](#_Toc199609225)

[Figure S4 5](#_Toc199609226)

[Table S1 6](#_Toc199609227)

[Table S2 7](#_Toc199609228)

#
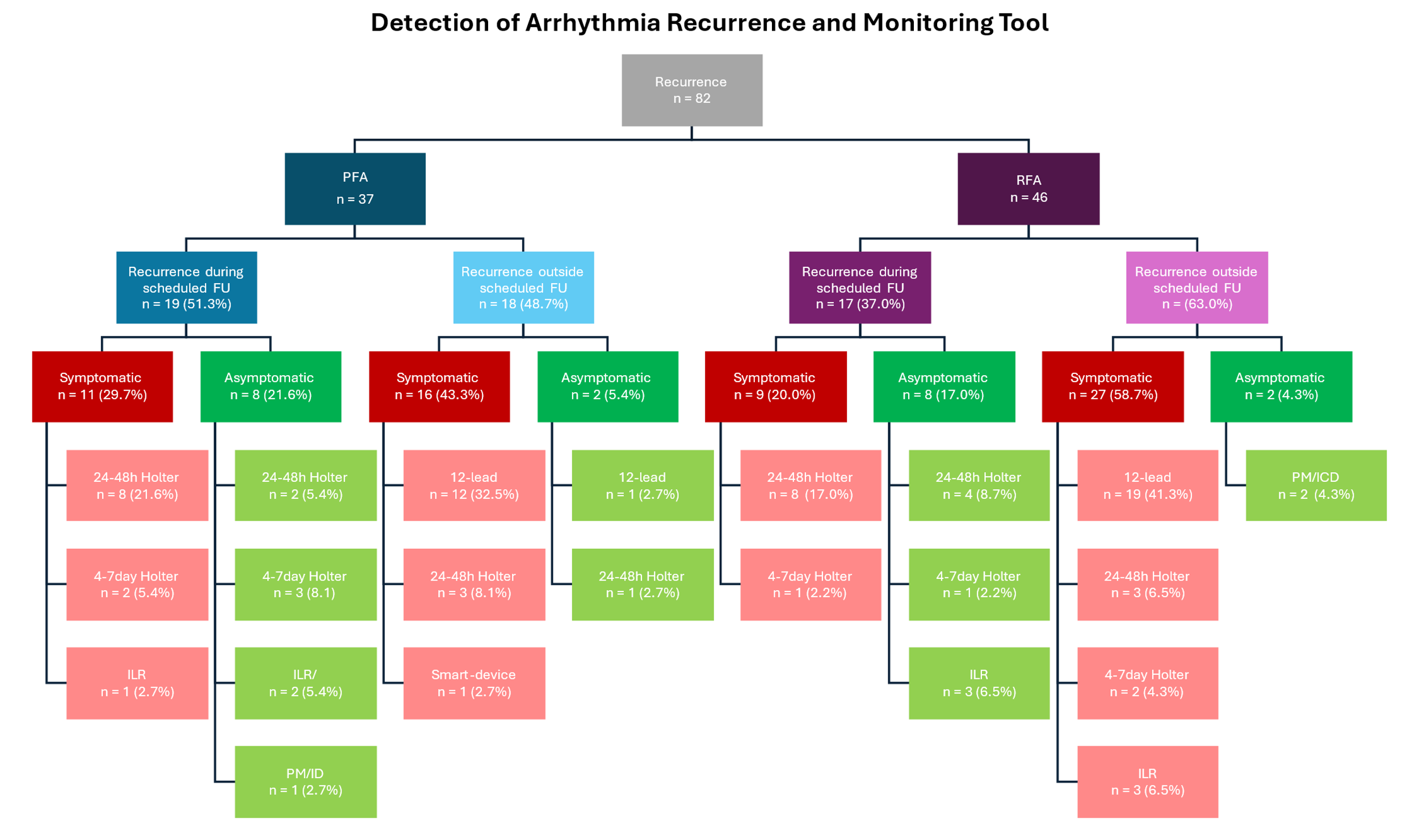
Figure S1

**Figure S1:** Flowchart illustrating detection of arrhythmia recurrence, and the rhythm monitoring modality used, stratified by ablation modality. FU = Follow-up; ILR = Implantable loop recorder; PFA = Pulsed-field ablation; RFA; Radiofrequency ablation.

# Figure S2


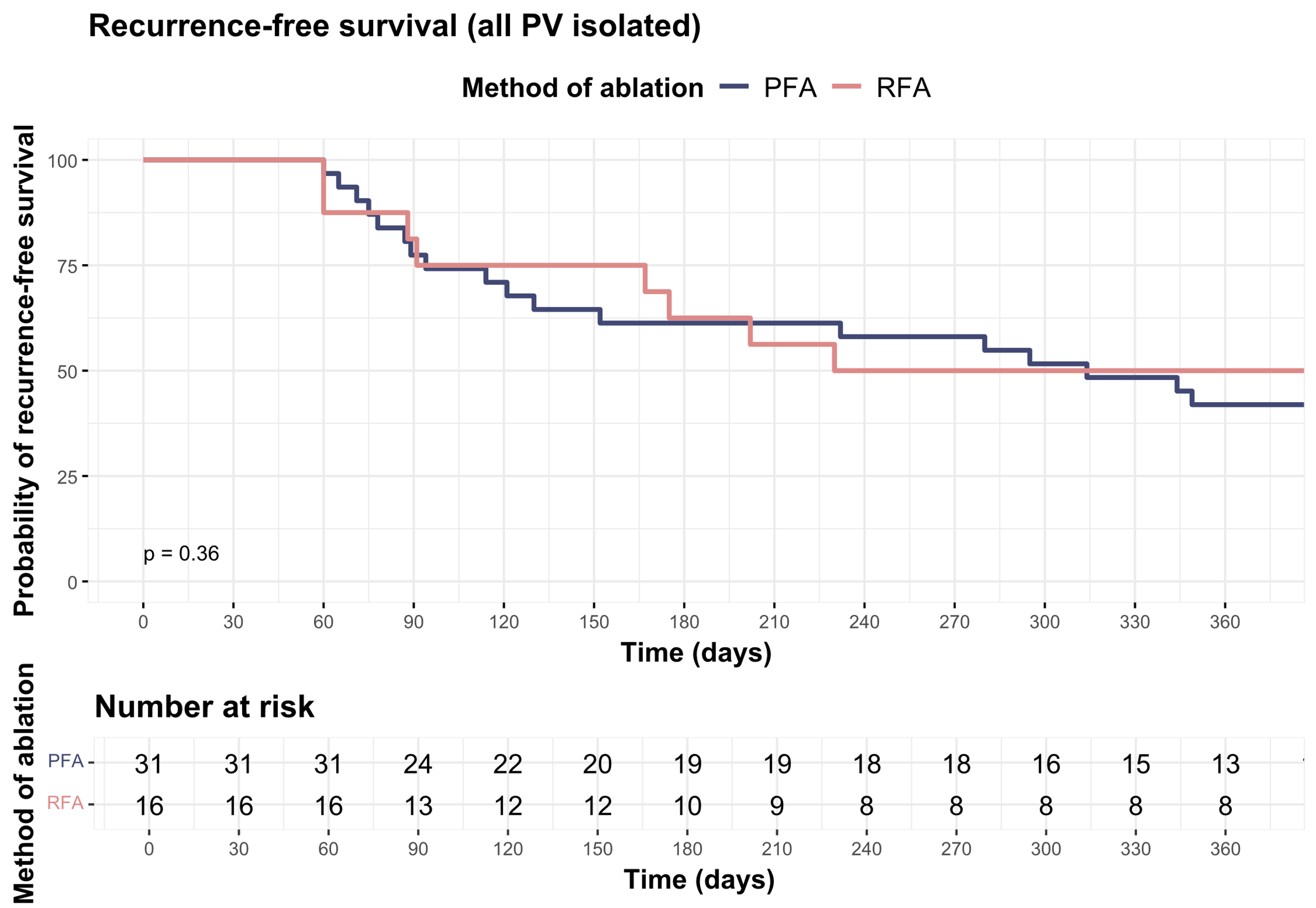


**Figure S2**: Subgroup analysis of patients with persistent isolated PVs after index ablation: Kaplan-Meier curve comparing the probability of recurrence-free survival over 1 year between the PFA and RFA groups. AF = Atrial fibrillation; PFA = Pulse-field ablation; PV = Pulmonary vein; RFA = Radiofrequency ablation.

# Figure S3


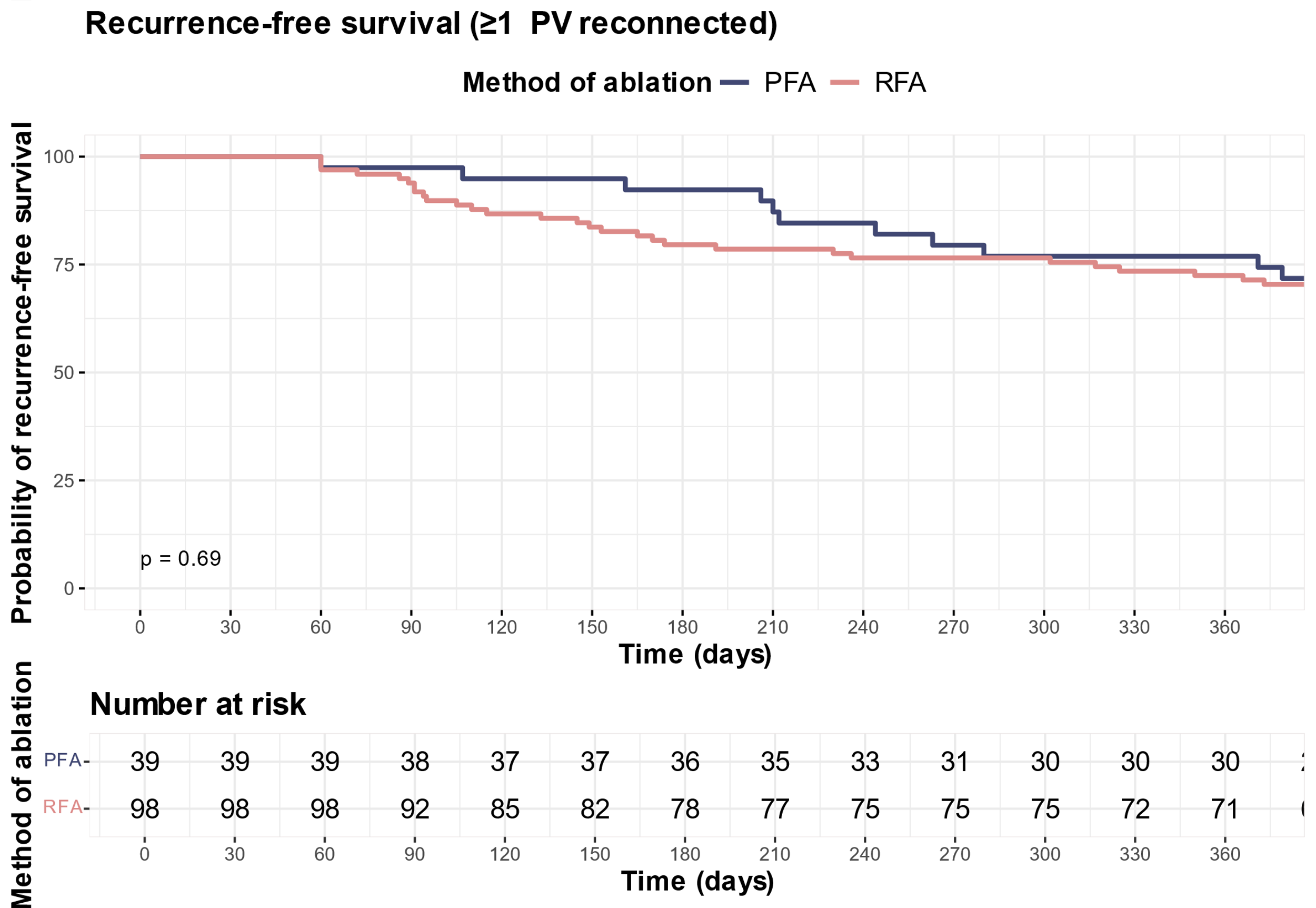


**Figure S3**: Subgroup analysis of patients at least one reconnected PV after index ablation: Kaplan-Meier curve comparing the probability of recurrence-free survival over 1 year between the PFA and RFA groups. AF = Atrial fibrillation; PFA = Pulse-field ablation; PV = Pulmonary vein; RFA = Radiofrequency ablation.

#
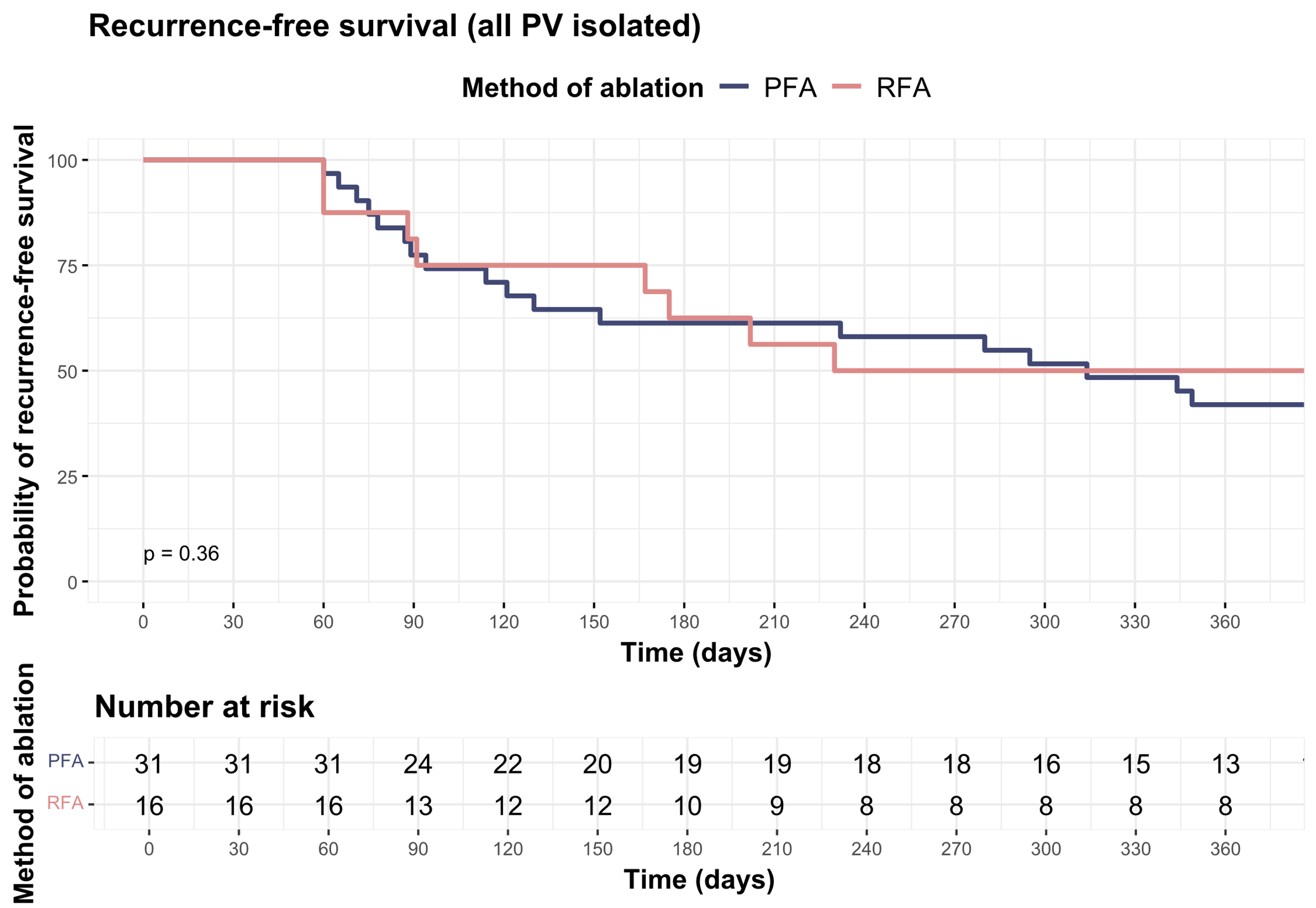
Figure S4

**Figure S4**: Kaplan-Meier curve for subgroup analysis of patients with persistent AF, comparing the probability of recurrence-free survival over 1 year between the PFA (n=43) and RFA groups (n=43). AF = Atrial fibrillation; PFA = Pulse-field ablation; RFA = Radiofrequency ablation.

|  | **Overall** N = 185 | **PFA**  N = 71 | **RFA**  N = 114 | **p-value** |
| --- | --- | --- | --- | --- |
| **Patients with continuous rhythm monitoring** | 16 (9%) | 7 (10%) | 9 (8%) | 0.644 |
| **Patients with recurrence** | 83 (45%) | 37 (52%) | 46 (40%) | 0.118 |
| **Timing of recurrence detection** |  |  |  | 0.188 |
| during FU | 36 (43%) | 19 (51%) | 17 (37%) |  |
| outside FU | 47 (57%) | 18 (49%) | 29 (63%) |  |
| **Symptomatic recurrence** |  |  |  | 0.576 |
| symptomatic | 63 (76%) | 27 (73%) | 36 (78%) |  |
| asymptomatic | 20 (24%) | 10 (27%) | 10 (22%) |  |
| **Used tool for recurrence detection** |  |  |  | 0.715 |
| 12-lead | 32 (39%) | 13 (35%) | 19 (42%) |  |
| 24-48h Holter | 29 (35%) | 14 (38%) | 15 (33%) |  |
| 4-7day Holter | 8 (10%) | 5 (14%) | 3 (7%) |  |
| ILR | 9 (11%) | 3 (8%) | 6 (13%) |  |
| PM/ICD | 3 (4%) | 1 (3%) | 2 (4%) |  |
| Smart device | 1 (1%) | 1 (3%) | 0 (0%) |  |

# Table S1

**Table S1**: Recurrence characteristics and monitoring modalities. FU = Follow-up; ILR = Implantable loop recorder; PFA = Pulsed-field ablation; RFA; Radiofrequency ablation.

# Table S2

|  | **Overall**  N = 86 | **PFA**  N = 43 | **RF**  N = 43 | **p-value** |
| --- | --- | --- | --- | --- |
| **Baseline characteristics** | | | | |
| **Age, years** | 69 [61 - 75] | 69 [63 - 76] | 69 [61 - 75] | 0.589 |
| **Sex (female)** | 19 (22%) | 9 (21%) | 10 (23%) | 0.795 |
| **BMI, kg/m^2^** | 27 [25 - 31] | 28 [25 - 33] | 27 [23 - 30] | 0.128 |
| **CHADS2-VASc Score** | 2 [1 - 3] | 2 [1 - 3] | 3 [1 - 4] | 0.582 |
| **LA diameter, mm** | 43 [39 - 46] | 44 [40 - 47] | 43 [38 - 46] | 0.157 |
| **LVEF %** | 55 [50 - 59] | 54 [48 - 58] | 55 [51 - 61] | 0.189 |
| **Coronary artery disease** | 9 (10%) | 6 (14%) | 3 (7%) | 0.483 |
| **Hypertension** | 56 (65%) | 31 (72%) | 25 (58%) | 0.175 |
| **Hypercholesterinemia** | 27 (42%) | 18 (53%) | 9 (29%) | 0.051 |
| **Diabetes** | 4 (5%) | 3 (7%) | 1 (2%) | 0.616 |
| **Smoking history** | 39 (49%) | 24 (60%) | 15 (38%) | 0.056 |
| **Procedural characteristics** | | | | |
| **Procedure duration, min** | 64 [51 - 78] | 62 [50 - 72] | 66 [55 - 98] | 0.137 |
| **LA Dwell time, min** | 47 [32 - 60] | 43 [33 - 55] | 54 [33 - 70] | 0.055 |
| **Fluoroscopy time, min** | 8 [5 - 12] | 10 [7 - 12] | 7 [4 - 12] | **0.020** |
| **Fluoroscopy dose, Gycm^2^** | 550 [341 - 1,091] | 614 [373 - 1,042] | 528 [266 - 1,191] | 0.619 |
| **Ablation Duration, min** | 18 [11 - 36] | 17 [12 - 24] | 23 [11 - 51] | 0.074 |
| **Mapping Duration, min** | 13 [11 - 20] | 14 [11 - 19] | 13 [10 - 24] | 0.883 |
| **Rhythm before ablation** |  |  |  | **0.012** |
| AF | 39 (46%) | 25 (60%) | 14 (33%) |  |
| AT | 12 (14%) | 2 (5%) | 10 (23%) |  |
| SR | 34 (40%) | 15 (36%) | 19 (44%) |  |
| **Ablation method of index PVI** |  |  |  | **0.019** |
| Cryo | 26 (30%) | 18 (42%) | 8 (19%) |  |
| RF | 60 (70%) | 25 (58%) | 35 (81%) |  |

| **Number of reconnected veins** |  |  |  | 0.003 |
| --- | --- | --- | --- | --- |
| **0** | 27 (31%) | 21 (49%) | 6 (14%) |  |
| **1** | 26 (30%) | 12 (28%) | 14 (33%) |  |
| **2** | 25 (29%) | 8 (19%) | 17 (40%) |  |
| **≥3** | 8 (9%) | 2 (5%) | 6 (14%) |  |
| **Reconnected LIPV** | 15 (17%) | 5 (12%) | 10 (23%) | 0.155 |
| **Reconnected LSPV** | 22 (26%) | 9 (21%) | 13 (30%) | 0.323 |
| **Reconnected RIPV** | 34 (40%) | 8 (19%) | 26 (60%) | <0.001 |
| **Reconnected RSPV** | 31 (36%) | 12 (28%) | 19 (44%) | 0.116 |
| **Hs-cTnT 1 prior to PVI, µg/l** | 11 [8 - 17] | 11 [8 - 17] | 10 [8 - 16] | 0.462 |
| **Hs-cTnT 1 day after PFA, µg/l** | 504 [216 - 876] | 793 [519 - 1,005] | 324 [141 - 449] | <0.001 |
| **Recurrence** | | | | |
| **Recurrence** | 48 (56%) | 26 (60%) | 22 (51%) | 0.385 |
| **Type of Recurrence** |  |  |  | 0.938 |
| **AF** | 33 (69%) | 18 (69%) | 15 (68%) |  |
| **AT / AFlu** | 15 (31%) | 8 (31%) | 7 (32%) |  |
| **Redo PVI** | 19 (22%) | 11 (26%) | 8 (19%) | 0.436 |
| **Median follow-up, days** | 488 [206 - 787] | 398 [210 - 700] | 539 [174 - 912] | 0.331 |

**Table S2**: Subgroup analysis of patients with persistent AF: patient characteristics, procedural characteristics, and recurrence of atrial arrhythmia in patients undergoing PFA and RFA. P-values were calculated using 3-sample test for equality of proportion, Pearson's Chi-squared test, Fisher's exact test or Wilcoxon rank sum test, as appropriate. AF = Atrial fibrillation; AT = Atrial tachycardia; Cryo = Cryoballoon ablation; LA = Left atrial; LIPV = Left inferior pulmonary vein; Hs-cTnT = high-sensitive cardiac troponin T; LSPV = Left superior pulmonary vein; PFA = Pulsed-field ablation; RFA = Radiofrequency ablation; RIPV = Right inferior pulmonary vein; RSPV = Right superior pulmonary vein; SR = Sinus rhythm.
